# The Effect of High Risk Pregnancy on Duration of Neonatal Stay in Neonatal Intensive Care Unit

**Published:** 2014-07-29

**Authors:** Narges Afrasiabi, Parisa Mohagheghi, Majid Kalani, Gholam Mohades, Zahra Farahani

**Affiliations:** 1 Maternal, Fetal and Neonatal Research Center, Tehran University of Medical Sciences; 2 Akbarabadi Hospital, Iran University of Medical Sciences; 3 Tehran University of Medical Sciences, Tehran, Iran

**Keywords:** Pregnancy, Complication, High Risk Pregnancy, NICU, Intensive Care Units, Neonatal

## Abstract

***Objective:*** High risk pregnancies increase the risk of neonatal mortality and morbidity. In order to identify the influence of pregnancy complications on the period of neonatal stay in Neonatal Intensive Care Units (NICUs), an analysis has been carried out in our center.

***Methods:*** In a cross-sectional-descriptive analytical study, the data including NICU length of stay was gathered from 526 medical records of neonates. We also assessed their maternal complications such as premature rapture of membranes (PROM), urinary tract infection (UTI), preeclampsia, oligohydramnios, and twin/triplet pregnancy. Finally we analyzed the relation between variables by SPSS statistics software version 19. The level of significance was considered *P*<0.05.

***Findings:*** 37 of 526 neonatal medical records were excluded. Of the 489 babies hospitalized in NICU for 1 to 54 days; 28.42% born were preterm, 308 with birth weight <2500 gram and 170 with birth weight between 2500 and 4000 gram. There was a significant relation between length of neonatal NICU stay and maternal PROM (*P*=0.001), preeclampsia (*P*=0.01), UTI (*P*=0.02), multiple gestation (*P*=0.03), and oligohydramnios (*P*=0.003). We found a positive correlation between numbers of gestation and length of NICU stay (*P*=0.03). A positive correlation existed between neonatal complication and length of NICU stay (*P*<0.001).

***Conclusion:*** By increasing maternal health level and prenatal care services, neonatal outcome can be improved and length of stay in NICUs decreased.

## Introduction

High risk pregnancy refers to pregnancy accompanied by factors which increase the risk of neonatal mortality and morbidity. Based on statistics 10-20% of pregnancies are reported as high risk pregnancies^[^^1^^]^. Neonatal state of health has a considerable effect on future health and life. Since neonate`s immune system and other organs in preterm neonate are not developed completely, they are at risk of many threats resulting neonatal admission in neonatal intensive care units (NICUs) for a short or long time in the first month of life^[^^2^^]^.

 Previous studies showed that preterm and late preterm babies had an increased risk ratio of both acute and long term morbidities and such complications affect the length of NICU stay compared with term infants^[^^3^^,^^4^^]^. Premature rapture of membranes (PROM) is another major clinical complication that is often related to high rates of neonatal morbidity and mortality^[^^5^^]^. Some studies have also indicated increased rates of morbidity and mortality in late preterm infants of women with gestational hypertension or preeclampsia. More neonatal intensive care unit admissions, hypoglycemia, respiratory distress, and re-hospitalization were seen in these children^[^^6^^]^. Maternal gestational diabetes mellitus (GDM) is another clinical complication associated with increased prenatal morbidity. Neonatal hyperglycaemia-related events, such as hypoglycemia, respiratory distress syndrome (RDS), hyperbilirubinemia, congenital anomaly, large for gestational age (LGA), primary cesarean section, polyhydramnios, preterm delivery, admission to NICU >24 h, are more frequent in mothers with GDM. NICU admission is reported in 29% of GDM and 40% in type 2 diabetes mellitus (DM) pregnancies^[^^7^^-^^9^^]^. Longer NICU stay was seen in pregnancies complicated by concurrent existence of hypertension and diabetes^[^^10^^]^.

 The aim of this study was to identify the influence of pregnancy complications on the period of neonatal length of stay in NICU. The provision of neonatal intensive care unit for complicated newborns is a great burden on the health care system. In the USA the cost of preterm babies care has been estimated about $8 billion annually^[^^11^^]^. Although there have been many studies looking at those factors resulting in newborn’s admission in NICU, but there is little information regarding the effects of maternal complications on duration of neonatal hospitalization. Such investigation would be beneficial for health organizations to access plans and proper strategies to decrease high risk pregnancies and consequently neonatal NICU hospitalization period. As a result, these strategies not only would be effective in preventing poor antenatal outcome, but also decrease the health system cost significantly.

## Subjects and Methods

A cross-sectional-descriptive analytical study was carried out in the NICU ward of Akbarabadi Hospital in Tehran, during 6 months in 2011. All data were gathered from neonates and their mothers’ medical records. Of 526 NICU admitted children, 37 newborns were excluded due to transfer from other centers (lack of data). The target population consisted of 489 newborns, admitted in the NICU for at least one day. Neonatal gestational age, sex, newborns’ problems, the length of neonatal stay at NICU (days) were recorded in a check list. At the same time we assessed maternal obstetric medical records and gathered data for mothers' complications (PROM, preeclampsia, urinary tract infection (UTI), GDM, vaginal bleeding, addiction). Finally we evaluated statistically the effects of these complications on neonates' admission period in NICU. The software package SPSS version 19 was used to perform the statistical analysis. The t-test, Chi square, regression and ANOVAs analysis were applied where applicable. The level of significance was considered *P*<0.05. 

 Patients’ data were handled confidentially and as no intervention was performed in our study, we did not ask for patients consent. Ethics approval for the study was obtained from the institutional review board of Tehran University of Medical Sciences.

## Findings

Thirty seven of 526 neonatal medical records were excluded. Of the remaining 489 babies hospitalized at NICU for 1 to 54 days (mean 7.9 days), 281 were males, and 28.42% born preterm. 308 newborns weighed <2500 g at birth and 170 neonates had a birth weight between 2500 and 4000 gr. 80.5% were singletons. 

 Of 489 mothers, 150 had PROM, 4.1% GDM, 1.84% UTI, 13.91% preeclampsia, 12.1% vaginal bleeding, 10.4% needed assisted reproductive techniques (ART), and in 1.6% drug abuse was recorded. Some demographic neonatal and maternal characteristics are shown in Table 1. Among neonates admitted to NICU, 322 cases (65.8%) had RDS, 20 cases (1.4%) showed seizures, 5 (1%) had sepsis and 1 (0.2%) had NEC. Twenty-two percent of admitted neonates died mostly (9%) due to RDS. 

**Table1:** Demographic characteristics of admitted neonates and their mothers in NICU

**Variable **		**Frequency (%)** **n = 489**
**Gender **	male	281 (57.5)
	female	208 (42.5)
**Gestational age (week) **	<37	132 (26.9)
	>37	357 (73.1)
**Birth weight (gram)**	<2500	308 (63.0)
	2500-4000	170 (34.8)
	>4000	11 (2.2)
**Expired newborns in NICU days**		108 (22.1)
**Complications **	Twin	74 (14.9)
	Triplet	22 (4.5)
	PROM	150 (30.7)
	Gestational diabetes mellitus	20 (4.1)
	Urinary tract infection	9 (1.8)
	Preeclampsia	68 (13.9)
	Drug addiction	8 (1.1)
	Vaginal bleeding	59 (12.1)
	Assisted reproductive techniques	51 (10.4)
	Oligohydramnios	28 (5.7)

NICU: neonatal intensive care units; PROM: Premature rapture of membranes

 There was a significant relation between duration of neonatal NICU length of stay and maternal PROM (*P*=0.001), preeclampsia (*P*=0.01), UTI (*P*=0.02), multiple gestation (*P*=0.03), and oligohydramnios (*P=*0.003) (Table 2). A positive correlation between neonatal complications and length of stay in NICU (*P*<0.001) was noticeable. The longest and shortest periods of NICU hospitalization belonged to neonates with prematurity and necrotizing enterocolitis (9.02 vs 5.10 days). Moreover, the highest mean NICU admission period were seen in premature neonates with both RDS and neonatal seizure symptoms (38 days). 

 We also found a positive correlation between numbers of gestation and length of NICU stay (*P*=0.03). Length of stay at NICU for singletons was 7.53 while in twin and triple gestations it was 9.52 and 9.54 days, respectively.

 Mortality rate in newborns whose mothers’ pregnancy was complicated by DM, vaginal bleeding and pregnancies following ART were significantly more than others (*P*=0.02, *P*=0.001, *P*=0.04, respectively). 

**Table 2:** Comparison of length of stay in NICU of neonates of mothers with and without complications

**Groups**		**Days in NICU ** **(mean)**	***P*** ** value**
**Premature Rapture of Membranes **	Yes	10.8	0.001
	No	6.09	
**Gestational Diabetes Mellitus **	Yes	8.45	>0.05
	No	7.92	
**Urinary Tract Infection**	Yes	5.22	0.02
	No	7.99	
**Preeclampsia**	Yes	10.82	0.01
	No	7.47	
**Drug addiction**	Yes	7.62	>0.05
	No	7.94	
**Vaginal bleeding**	Yes	7.42	>0.05
	No	8.01	
**Assisted Reproductive Techniques**	Yes	7.87	>0.05
	No	8.52	
**Oligohydramnios**	Yes	11.78	0.003
	No	7.70	

## Discussion

Neonatal mortality and morbidity is common following complicated pregnancies. This study examined whether high risk pregnancies could influence neonatal outcome and length of NICU hospitalization. 

 We found that maternal PROM, preeclampsia, oligohydramnios, UTI, and multiple gestations had increased duration of NICU stay. Previous studies have shown that PROM and preterm birth after PROM are associated with small-for-gestational age and low birth weight newborns. In addition the high rate of cesarean sections, chorioamnionits, fetal distress and placental accidents were seen more frequently in these groups^[^^5^^]^. PROM accounts for 25-40% of all preterm deliveries that increase the risk of neonatal morbidity by 75%. In addition, improvement in survival may be associated with adverse long term sequels needing more treatment and NICU hospitalization^[^^11^^]^. There is no doubt that pregnancies complicated with hypertension have higher rates of neonatal morbidity than normotensive pregnancies. Many studies have indicated that mothers with preeclampsia have increased rates of small for gestational age (SGA) infants^[^^3^^]^. Preeclampsia increases the rate of induction at each gestational week and cesarean section, both of them resulting in SGA infants’ birth with higher incidence of complications like RDS. Some forms of IUGR etiologically are linked to preeclampsia and its placental dysfunction. Hypertensive disorders in pregnancy also raise the incidence of NICU admission at 35, 36, and 37 weeks of gestation and longer neonatal stay. The gestational week of delivery rather than severity of complication had greater role on NICU admission and total stay. Even IUGR neonates from preeclamptic mothers were at a higher risk of NICU stay 7 days or more than unexplained IUGR neonates^[^^6^^,^^12^^]^. 

 Oligohydramnios, systemic or regional infection (such as urinary tract infection) and multi fetal pregnancy are predisposing factors for preterm birth. Both lower gestational age and birth weight have adverse roles on morbidity following preterm birth. In developed countries, preterm birth is responsible for 75% of neonatal morbidity including neurodevelop-mental complication, pulmonary disease, and visual problem. Moreover singletons survive better in comparison with twins. The incidence of intraventricular hemorrhage (IVH) and RDS are higher in preterm twins than in singletons^[^^11^^]^. 

 We also found that the multiple gestation affects length of NICU stay. In recent decades multiple births have increased due to increased employing of assisted reproductive techniques. Multiple pregnancies increase the rate of stillbirth, early and late neonatal mortality and morbidity. The main cause is related to prematurity, LBW and intra partum complications. In a tertiary referral center, 90% of triplets with gestational age <32 weeks were admitted in neonatal unit. Cerebral palsy in triplets and twins occurs 47 and 8 times more compared to singletons. Triplets and high multiple gestations stayed longer in neonatal unites (51 days) than twins (40 days)^[^^13^^,^^14^^]^. 

 We found that the highest mean length of NICU stay was seen in neonates with RDS and neonatal seizure, also correlation between neonatal mortality and RDS was noticeable. Our results were consistent with other studies. An investigation has demonstrated higher morbidity rate in preterm neonates at NICUs. Moreover RDS is the main common cause of neonatal mortality. Of infants born at 30-34 weeks, 28% had an acute lung disorder^[^^15^^]^. Late preterm birth (34-36 weeks) with higher risk of RDS accounts for 71.7% NICU admissions in USA^[^^16^^]^. Mortality rate and the risk of RDS are high among 26-29 weeks SGA neonates. The effect of SGA and prematurity may influence events resulting in increasing mortality and neonatal morbidity rates. Fetal hypoxemia, nutrient restriction and altered endocrine milieu could be affecting factors^[^^17^^]^. In addition, prevalence of neonatal seizures is around 1-3 per 1000 live births and more frequent in preterm population. Seizures increase neurological and developmental morbidity. In preterm babies 40% of seizures result from hypoxic-ischemic events. In a cohort study, 28% of neonatal seizure survivors had poor outcome like hypoxic-ischemic encephalopathy, focal infarction, subarachnoid hemorrhage, metabolic disorder, meningitis, or congenital brain malformation. Seizure with long adverse outcome increases neonatal need for prolonged medical treatment and clinical attention^[^^18^^]^. 

 Finally our analysis revealed that mortality rate in newborns whose mothers pregnancy was complicated by GDM, vaginal bleeding, and ART was higher than in others. We speculated that all these conditions could be predisposing factors for preterm births, SGA and VLBW infants resulting rise in neonatal mortality rate. Low birth weight was common in our study population. Our results were compatible with other reported studies; neonatal mortality rate was higher in mothers with vaginal bleeding and third trimester complications^[^^19^^,^^12^^]^. In an investigation carried out in Nigeria, among 87 neonatal deaths; SGA and LBW infants had greatest portion (21.7% and 20.1% respectively)^[^^20^^]^. Furthermore studies showed that 85% of transferred embryos by ART could not produce a live birth^[^^21^^]^. ART also increases obstetric and perinatal adverse outcomes like perinatal death, chromosomal abnormalities, low birth weight, preterm labor, GDM, placental accidents and preeclampsia^[^^22^^]^.

*Limitation:* In our study we considered only a few obstetrical complications like preeclampsia, vaginal bleeding, GDM, oligohydramnios, and preterm birth. We recommend to survey the effect of other direct and indirect obstetric morbidities in future studies.

## Conclusion

Maternal complication can influence neonatal outcome significantly. By increasing the maternal health level and prenatal care services, we can improve neonatal outcome and decrease length of stay in NICU.
